# Editorial: Deep Learning in Biological, Computer, and Neuromorphic Systems

**DOI:** 10.3389/fncom.2019.00011

**Published:** 2019-03-08

**Authors:** Evgeniy Bart, Jay Hegdé

**Affiliations:** ^1^Palo Alto Research Center, Palo Alto, CA, United States; ^2^Department of Neuroscience and Regenerative Medicine, James and Jean Culver Vision Discovery Institute, The Graduate School, and Department of Ophthalmology, Medical College of Georgia, Augusta University, Augusta, GA, United States

**Keywords:** perceptual learning, convolutional and recurrent neural networks, implicit learning, statistical learning, artificial neural network

Deep learning is a branch of machine learning in which statistical representations of the input data, as opposed to task-specific algorithms, are learned. Deep learning may be supervised, unsupervised, or semi-supervised (Lecun et al., 2015; Schmidhuber, 2015; Goodfellow et al., 2016). Deep learning techniques are behind many impressive recent successes of machine learning; for example, a deep learning machine recently beat the world champion at the game of *Go* (Silver et al., 2016), a highly significant achievement that had remained out of reach for the past 50 years.

The current Research Topic provides a useful overview of the capabilities and the applications of deep learning and of attempts to elucidate some of its general principles.

Many of the applications in the current Research Topic focus on various aspects of neurology and neuroscience. For example, Liu et al. use deep learning to distinguish patients with Alzheimer's disease from healthy controls using PET images; Wang and Ke use an artificial neural network (ANN) to identify epileptic seizures in EEG recordings; Hegdé and Bart use deep learning and deep synthesis to generate artificial but naturalistic mammogram images for psychophysics experiments; and Zhang et al. attempt to perform “mind reading” by reconstructing an image being viewed by a subject based on the subject's MRI recording.

Additional papers address the underpinnings of deep learning as an approach. For example, Wan and Song investigate ways to add hints to a network to improve its performance, and Thiele et al. propose a spiking deep network architecture that is suitable for online deep learning. Finally, Bart and Hegdé investigate the explainability of decisions learned in a weakly guided manner, an issue that is relevant to both biological and artificial learning systems.

## Parallels With Human Statistical Learning

Many deep learning approaches were inspired by contemporary studies of the brain function. These machine learning studies have directly adopted—quite reasonably (except perhaps from the perspective of the most parochial practitioners of pedantic turf battles)—the relevant neurobiological terminology: The building blocks of a deep learning machine are called “neurons,” and they simulate a few basic properties of biological neurons, such as the ability to integrate inputs from a few simulated “synapses” and, under the right conditions, to simulate “firing” by outputting appropriate signal into outgoing connections.

Thus, it is not surprising that there are striking, if broad, parallels between what can be learned by machine and biological systems. Humans and many other species can learn detailed, sophisticated representations of spatial and temporal patterns in visual, auditory, and tactile data (Santolin and Saffran, 2018) with limited explicit guidance. Supervised and unsupervised statistical learning in human infants is also well-established (Saffran and Kirkham, 2018).

However, there is no established terminology for these biological equivalents of artificial deep learning. Perceptual learning of statistical patterns in semi-supervised or unsupervised settings is sometimes referred to as “implicit learning” because, in this case, the observer learns the representations of the input data without being told what to learn (Seger, 1994; Hegdé et al., 2008). It is self-evident that these instances of statistical learning by biological systems are functionally analogous to deep learning by machine systems, in that statistical representations of input data are learned in both cases. However, implicit learning can also refer to cases where the learning is not statistical *per se* (see Seger, 1994). Thus, there is currently no term for the biological analog of machine deep learning. We therefore advocate, with a nod to the aforementioned adoption of neurobiological terms in the machine learning literature, that such learning by biological systems be simply referred to as “biological deep learning”.

It is clear that the implementation-level details of deep learning must, of necessity, be different between the two types of systems. However, whether and to what extent the underlying *computations* are similar in the two cases remains to be characterized.

## Future Directions

Despite impressive recent progress, numerous issues pertaining to deep learning still remain unresolved. Here, we highlight a selected few of these issues.

Perhaps one of the central issues in the field is the question of how deep learning works in machines (Goodfellow et al., 2016) and in neural systems (Connolly, 2019). It is also currently impossible to characterize the general conditions under which deep learning is expected to succeed or fail, in either biological or machine learning context. Despite numerous successes, this leads to significant limitations of existing deep learning methods (e.g., Szegedy et al., 2014) and hinders future progress. A specific examplar question along these lines is whether deep learning exhibits domain generality but modality specificity, as some other types of statistical learning (see Frost et al., 2015).

Another important question is the extent and mechanisms of interaction between the deep learning systems with other systems of learning. These are likely to take place in biological systems, as not all learning in biology qualifies as deep learning. Elucidating these interactions could allow generalizing them to machine learning systems, where they could be used, for example, to incorporate useful prior knowledge (such as the laws of physics).

Finally, applications of deep learning become increasingly impactful. These include self-driving cars, personal assistants, and, in the not-so-distant future, deep learning machines embedded in biological systems (such as smart medical devices or robots that learn what their clients want). Since they have the potential to significantly affect daily lives of millions or even billions of people, it becomes particularly important to characterize the behavior of these deep learning systems. One necessary characterization is the ability to explain the system's behavior in ways humans can understand. Another, perhaps more important, is the ability to prove certain properties or invariants of the system's behavior, such as that the given system will always obey certain laws or will never exhibit certain behaviors, akin to Asimov's Three Laws of Robotics.

We hope that future interdisciplinary collaboration will help resolve these issues.

## Author Contributions

Both authors listed have made a substantial, direct and intellectual contribution to the work, and approved it for publication.

### Conflict of Interest Statement

The authors declare that the research was conducted in the absence of any commercial or financial relationships that could be construed as a potential conflict of interest.
